# An overview of low-oxygen sensing and flooding responses of tomato

**DOI:** 10.1093/jxb/eraf203

**Published:** 2025-05-15

**Authors:** Niels Eerdekens, Elif Nur Kabak, Batist Geldhof, John Vaughan-Hirsch, César Antonio Chavez, Francesco Mignolli, Maria Laura Vidoz, Bram Van de Poel

**Affiliations:** Division of Crop Biotechnics, Department of Biosystems, University of Leuven, Leuven 3001, Belgium; Leuven Plant Institute, Leuven 3001, Belgium; Division of Crop Biotechnics, Department of Biosystems, University of Leuven, Leuven 3001, Belgium; Leuven Plant Institute, Leuven 3001, Belgium; Leuven Plant Institute, Leuven 3001, Belgium; Micro- and Nanosystems (MNS), Department of Electrical Engineering (ESAT), University of Leuven, Leuven 3001, Belgium; Division of Crop Biotechnics, Department of Biosystems, University of Leuven, Leuven 3001, Belgium; Leuven Plant Institute, Leuven 3001, Belgium; Instituto de Botánica del Nordeste (IBONE), UNNE-CONICET, Corrientes 3400, Argentina; Instituto de Botánica del Nordeste (IBONE), UNNE-CONICET, Corrientes 3400, Argentina; Facultad de Ciencias Agrarias, Universidad Nacional del Nordeste (UNNE), Corrientes 3400, Argentina; Instituto de Botánica del Nordeste (IBONE), UNNE-CONICET, Corrientes 3400, Argentina; Facultad de Ciencias Agrarias, Universidad Nacional del Nordeste (UNNE), Corrientes 3400, Argentina; Division of Crop Biotechnics, Department of Biosystems, University of Leuven, Leuven 3001, Belgium; Leuven Plant Institute, Leuven 3001, Belgium; University of Malaga, Spain

**Keywords:** Adventitious roots, aerenchyma, epinasty, flooding, hormones, low-oxygen signaling, tomato, waterlogging

## Abstract

Tomato (*Solanum lycopersicum*) is a globally significant and widely consumed vegetable crop. However, the productivity of tomato cultivation is increasingly threatened by flooding events, which are predicted to escalate in both frequency and severity due to climate change. During waterlogging, plants experience acute hypoxic stress, which can be lethal if prolonged. This review examines the mechanisms by which plants sense and signal low-oxygen stress, with a focus on the role of group VII ethylene response factors and the N-degron pathway, including their regulation. A comparative analysis of these low-oxygen signaling pathways between Arabidopsis and tomato reveals considerable conservation across species, although understudied in tomato. Furthermore, this review elucidates how hypoxia triggers various adaptation strategies in tomato. We highlight the physiological, morphological, metabolic, and hormonal responses, including modifications in plant transpiration and photosynthesis, the development of aerenchyma and adventitious roots, the induction of epinasty, and the reprogramming of energy metabolism. The review also provides insights into the hormonal signaling cascades that play a pivotal role in flooding stress responses. We aim to provide an in-depth understanding of how tomato plants deal with flooding-induced hypoxic stress. Additionally, we aim to provide insights that can be leveraged for breeding more flood-tolerant and climate-resilient tomato cultivars.

## Teaser

Flooding stress can severely impact tomato cultivation and productivity. This review explores various aspects of low-oxygen stress in tomato, including hypoxia signaling, hormonal regulation, and physiological and morphological responses.

## Introduction

Tomato is one of the most produced and consumed vegetables worldwide, with a global production of 186 Mt in 2023 (FAO, 2023). Various production systems exist, such as open field or plastic-covered soil-grown plantations, or greenhouse production systems with soilless substrates, either organic or not (Gould, 1992; Mattoo and Handa, 2017; Heuvelink, 2018). Greenhouse production systems have the advantage that crops are protected, and the climate is highly controlled, minimizing flooding risks. Although this system typically has a much higher yield per surface area, its energy consumption and greenhouse gas emission are much higher (Maureira *et al*., 2022).

Soil-grown production systems, on the other hand, are more vulnerable to flooding events, which include irregular and heavy rains, river inundations, and coastal floods. All these flooding events are projected to increase in frequency and severity due to climate change (Hunt, 2002; Bailey-Serres *et al*., 2012; Hirabayashi *et al*., 2013). As such, flooding events pose a serious threat to the agricultural productivity of tomato cultivation. The impact of these flooding events is dependent on both their duration and the extent to which the plant is submerged, with complete submergence being more severe than root-only submergence (waterlogging) (Colmer and Voesenek, 2009; Sasidharan *et al*., 2017; Loreti and Perata, 2020; Aslam *et al*., 2023; Dalle Carbonare *et al*., 2023).

Knowing how tomato plants sense and respond to flooding-induced low-oxygen stress is essential for breeding new varieties that are more suitable to grow in flood-prone areas. In this review, we summarize the latest findings in plant low-oxygen sensing and signaling, and we synthesize how tomato plants deal with flooding stress. We focus on the physiological, morphological, metabolic, and hormonal changes of tomato plants exposed to low-oxygen stress.

## Oxygen sensing and signaling in plants

The response to flooding is complex, with several interacting pathways playing major roles. Early signaling events largely result from slower gas diffusion under water, leading to changes in oxygen, carbon dioxide, nitric oxide (NO), and ethylene levels in submerged tissues. The exact concentration of these gases is highly dependent on the specific flooding conditions and tissues, and so there is a great diversity of responses (León *et al*., 2020). When reduced gas exchange during flooding is combined with reduced oxygen production by photosynthesis (e.g. in waterlogged roots or in shoots submerged in murky flood waters) and enhanced oxygen consumption by respiration and metabolism, plants rapidly experience low-oxygen stress (hypoxia). This ultimately leads to ATP and carbon starvation, compromising cellular function (Loreti and Perata, 2020). Furthermore, changes in cellular oxygen can also be deployed as an endogenous signal driving plant development (Hammarlund *et al*., 2020), including morphological responses (Daniel and Hartman, 2024). It is therefore crucial that oxygen signaling mechanisms are in place to drive transcriptional responses that allow adaptation to hypoxia, such as the switch to anaerobic respiration, or morphological and anatomical changes to facilitate plant survival and development.

### The N-degron pathway directs group VII ethylene response factor transcription factors during oxygen signaling

**Fig. 1.**
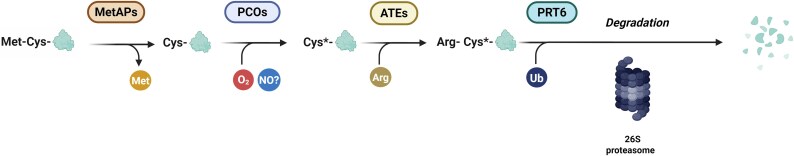
Schematic of plant oxygen sensing through the Arg/N-degron pathway in plants. Proteins with a terminal methionine–cysteine residue are targeted. The terminal Met is removed by methionine aminopeptidase (MetAP) followed by the oxidation of the terminal Cys by Plant Cysteine Oxidases (PCOs) in an oxygen-dependent manner. In the next step, Arginine transferase (ATE) conjugates an Arg residue to the protein, triggering ubiquitination by Proteolysis 6 (PRT6) which promotes the degradation of oxidized proteins by the 26S proteasome. Created in Biorender. Vanden Broeck, E. (2025) https://BioRender.com/ymc5n71.

Regulation of hypoxia responses is primarily dependent on the group VII ethylene response factor (ERF-VII) transcription factor family, which are stabilized during hypoxia (Gibbs *et al*., 2011; Licausi *et al*., 2011). The ERF-VIIs promote hypoxia tolerance by directly regulating the expression of hypoxia-responsive genes (HRGs) and are degraded via the oxygen-dependent branch of the N-degron pathway (the Arg/N-degron pathway) (Giuntoli and Perata, 2018). In this pathway, only proteins with an N-terminal methionine–cysteine (MC) residue are targeted for degradation (Fig. 1) (Bachmair *et al*., 1986). The first step is removal of the terminal methionine by METHIONINE AMINO-PEPTIDASE (MetAP) (Moerschell *et al*., 1990). The newly exposed terminal cysteine is then oxidized by PLANT CYSTEINE OXIDASEs (PCOs), arginylated by ARGINYL TRANSFERASES (ATEs), and ubiquitinated by the E3 ligase PROTEOLYSIS6 (PRT6), which targets the protein for degradation via the 26S proteasome (Garzón *et al*., 2007; White *et al*., 2017). The major limiting factor in this process is the availability of molecular oxygen, which is required for PCO activity (White *et al*., 2018; Triozzi *et al*., 2024). As oxygen levels drop during flooding, PCO activity is inhibited, preventing degradation of ERF-VIIs and consequently they activate expression of HRGs. These HRGs are involved in many adaptive responses to hypoxia, with several encoding enzymes involved in anaerobic metabolism (Giuntoli and Perata, 2018). NO is also required for a functional N-degron pathway, although the exact mechanism is unknown (Gibbs *et al*., 2011, 2014).

While the ERF-VIIs are likely to be the major N-degron substrates involved in hypoxia tolerance, two other substrates have so far been discovered in *Arabidopsis thaliana*, which are also degraded via the same N-degron pathway; namely VERNALISATION 2 (VRN2) and LITTLE ZIPPER 2 (ZPR2) (Gibbs *et al*., 2018; Weits *et al*., 2019). VRN2 is a subunit of the POLYCOMB REPRESSIVE COMPLEX 2 (PRC2), which promotes a repressive chromatin state by histone trimethylation (H3K27me3) at target sites (Förderer *et al*., 2016). The diversity of VRN2-PRC2 target sites is evidenced by the diversity of *vrn2* mutant phenotypes, in which processes such as seed development and dormancy, vascular development, somatic cell de-differentiation, vernalization, and hypoxia tolerance are affected (Gendall et al., 2001; Roszak and Köhler, 2011; Ikeuchi *et al*., 2015; De Lucas *et al*., 2016; Auge *et al*., 2017; Costa and Dean, 2019). ZPR2 is a transcription factor which regulates shoot apical meristem (SAM) activity by inhibiting expression of HD-ZIP III transcription factors. So far, only the ERF-VIIs, VRN2, and ZPR2 are known to be targets of the O_2_-dependent N-degron pathway. However, it is possible that other substrates, which could also contribute to hypoxia response, remain to be discovered.

The tissue-specific accumulation of N-degron pathway substrates, irrespective of environmental stimuli, points to zones of chronic hypoxia, known as hypoxic niches. These are often bulky organs which limit O_2_ diffusion, or regions of high metabolic activity where hypoxic signaling is required for normal development, such as in the SAM (Loreti and Perata, 2020; Weits *et al*., 2021). Both VRN2 and ZPR2 are stabilized in the SAM, and their localization is expanded during hypoxia (Gibbs *et al*., 2018; Weits *et al*., 2019; Labandera *et al*., 2021). VRN2 is also stabilized in other hypoxic niches throughout the plant, including root tips, lateral root meristem regions, and inflorescences (Labandera *et al*., 2021). Similarly, the activity of ERF-VIIs is associated with hypoxic niches, such as the root apical meristem region (Weits *et al*., 2021).

Alternatively, plant tissues can also experience transient changes in oxygen levels by means of cyclic or environmentally induced changes in photosynthesis or respiration that steer plant development (Herzog *et al*., 2023; Triozzi *et al*., 2024). In tomato, it has been shown that ripening fruit also experience internal hypoxia, which is mainly manifested in the locular gel and caused by diffusion resistances and high respiration rates during climacteric fruit ripening (Xiao *et al*., 2024).

### Regulation of N-degron substrate stability and group VII ethylene response factor activity

While N-degron targets are clearly stabilized by low O_2_ or NO conditions, there are several other factors influencing the activity of N-degron pathway components, and consequent substrate stability (White *et al*., 2017). VRN2, for example, is stabilized by cold treatment via repression of N-degron activity (Gibbs *et al*., 2018). While the exact mechanism remains unknown, it has been suggested that cold treatment leads to biochemical changes which mimic hypoxia, resulting in reduced N-degron activity. PCO activity can also be dampened in low-iron or zinc-rich conditions, as iron is required as a cofactor (Carbonare *et al*., 2019; Zubrycka *et al*., 2023). Ethylene, which accumulates during submergence, induces expression of the NO scavenger gene *PHYTOGLOBIN1* (*PGB1*), therefore dampening the N-degron pathway and leading to ERF-VII accumulation (Hartman *et al*., 2019). Recently, the protein BIG has also been implicated in enhancing the N-degron pathway, semi-redundantly with PRT6 (Zhang *et al*., 2024).

Besides manipulating N-degron activity, the activity of ERF-VIIs can also be directly modulated. This can be achieved by regulating the subcellular localization of ERF-VIIs. The ERF-VII RAP2.12, for example, is tethered to the plasma membrane via interaction with ACYL-CoA BINDING PROTEIN (ACBP) and is only released upon hypoxia due to low ATP-induced changes in acyl-CoA content (Schmidt *et al*., 2018; Zhou et al., 2020). Another way to regulate activity is by phosphorylation, which is required for optimal ERF-VII activity. One kinase responsible for this is the energy sensor TARGET OF RAPAMYCIN (TOR), which is active as long as there are suitable sugar reserves. Plants experiencing hypoxia typically have low sugar reserves, which reduces TOR activity and therefore ERF-VII activity is dampened (Kunkowska *et al*., 2023). This feedback loop regulates a proper balance between energy availability and hypoxia responses. Another mechanism is via the transcription factor HYPOXIA RESPONSE ATTENUATOR1 (HRA1), which directly binds and inhibits RAP2.12 (Giuntoli *et al*., 2014). *HRA1* is itself an HRG, and thus is transcriptionally up-regulated by ERF-VIIs, thereby forming a negative feedback loop.

### Homology between the Arabidopsis and tomato low-oxygen signaling pathway

The targets and responses of the O_2_-dependent branch of the N-degron pathway are well conserved in angiosperms (Reynoso *et al*., 2019; Hammarlund *et al*., 2020). Here, we compare Arabidopsis and tomato (Table 1). In *A. thaliana* (At) there are five *PCO* genes, two of which are transcriptionally up-regulated by ERF-VII activity (referred to as B-type). While the B-type *PCO* genes (*AtPCO1* and *AtPCO2*) are the best studied members, the A-type *PCO* genes also contribute to ERF-VII degradation in aerobic conditions, but are not transcriptionally regulated by hypoxia (Weits *et al*., 2023). Further in the N-degron pathway, there are two AtATEs (AtATE1 and AtATE2) and a single E3 ligase (AtPRT6). In tomato [*Solanum lycopersicum* (Sl)] there are six PCOs in total (three of each type), a single ATE, and two orthologs of PRT6 (Weits *et al*., 2023).

**Table 1.** Arg/N-degron pathway components and substrates in Arabidopsis and tomato and their regulation by hypoxia stress

Species	Gene name	Gene ID	Family	Transcriptional response to hypoxia*^a^*
**Arabidopsis**	AtPCO1	AT5G15120	PCO	Up-regulated*^b^*
	AtPCO2	AT5G39890	PCO	Up-regulated*^b^*
	AtPCO3	AT1G18490	PCO	
	AtPCO4	AT2G42670	PCO	
	AtPCO5	AT3G58670	PCO	
	AtATE1	AT5G05700	ATE	
	AtATE2	At3g11240	ATE	
	AtPRT6	At5g02310	PRT6	
	AtRAP2.2	AT3G14230	ERF-VII	
	AtRAP2.3	AT3G16770	ERF-VII	
	AtRAP2.12	AT1G53910	ERF-VII	
	AtHRE1	AT1G72360	ERF-VII	Up-regulated*^b^*
	AtHRE2	AT2G47520	ERF-VII	Up-regulated*^b^*
	AtVRN2	AT4G16845	VRN2	
	AtZPR2	AT3G60890	ZPR2	
	AtZPR1*^c^*	AT2G45450*^c^*	ZPR1*^c^*	
**Tomato**		Solyc02g087740	PCO	Up-regulated*^d^*
		Solyc02g067440	PCO	Up-regulated*^d^*
		Solyc03g113130	PCO	Up-regulated*^b,d^*
		Solyc07g055150	PCO	
		Solyc10g007990	PCO	
		Solyc03g114510	PCO	
		Solyc06g051760	ATE	
		Solyc09g010830	PRT6	
		Solyc10g084760	PRT6	
		Solyc01g065980	ERF-VII	
		Solyc06g063070	ERF-VII	Down-regulated*^e^*
		Solyc12g049560	ERF-VII	
		Solyc03g123500	ERF-VII	
		Solyc09g075420	ERF-VII	Up-regulated*^e^*
		Solyc03g093640*^c^*	VRN2*^c^*	
		Solyc01g091490	ZPR2	
		Solyc08g079690	ZPR2	
		Solyc08g007570	ZPR2	

^*a*^Data were analyzed from RNA-seq results obtained for shoot or root tissue during either hypoxia, submergence, or waterlogging treatments. An empty cell denotes no transcriptional response detected.

^*b*^ Tamura and Bono (2022).

^*c*^Genes which lack the destabilizing MC-amino residue and are therefore unlikely to be targets of the N-degron pathway.

^*d*^ Weits *et al*. (2023).

^*e*^ Safavi-Rizi *et al*. (2020).

As well as the N-degron pathway components, the three N-degron target families are represented in angiosperms. In Arabidopsis there are five *ERF-VII* genes, three of which (*AtRAP2.2*, *AtRAP2.3*, and *AtRAP2.12*) are probably transcriptionally up-regulated by the other two *(A*t*HRE1* and *AtHRE2*) (Bui *et al*., 2015). While all *ERF-VII* genes are stabilized by hypoxia, only *HRE1* and *HRE2* are transcriptionally up-regulated by hypoxia (Mustroph *et al*., 2009; Licausi *et al*., 2010). The other N-degron targets include AtVRN2 and AtZPR2 (Gibbs *et al*., 2018; Weits *et al*., 2019). In tomato, there are also five *SlERF-VII* genes, and three homologs of *AtZPR2* (Weits *et al*., 2023). There is also a single SlVRN2 which is probably not targeted by the N-degron pathway due to a lack of the MC-amino residue.

The N-degron targets have been most intensively studied in Arabidopsis, and those from other species may require experimental confirmation of their increased stability upon hypoxia treatment. However, the abundance of publicly available transcriptomics datasets provide insight into their regulation during hypoxia. For example, of the five *ERF-VII* regions of tomato, only one (*Solyc09g075420*) is transcriptionally up-regulated by hypoxia, similar to *AtHRE1* and *AtHRE2* (Safavi-Rizi *et al*., 2020; De Ollas *et al*., 2021). There is also one tomato *PCO* which is strongly up-regulated by hypoxia (*Solyc03g113130*), similar to *AtPCO1* and *AtPCO2*. These genes may have evolved to fulfill the same function in the different species. Ethylene, which accumulates during flooding, also influences expression of hypoxia-responsive genes, including those encoding ERF-VIIs. In Arabidopsis seedlings, *AtHRE1*, *AtRAP2.2*, and *AtRAP2.3* are up-regulated by ethylene treatment (Harkey *et al*., 2019). Similarly, in tomato leaves, four of the five ERF-VIIs (*Solyc01g065980*, *Solyc06g063070*, *Solyc03g123500*, and *Solyc09g075420*) are up-regulated by ethylene (Mohorović *et al*., 2024). In addition, a tomato homolog of *AtPGB1* (*Solyc07g008240*) is strongly up-regulated by ethylene treatment (Mohorović *et al*., 2024), suggesting that it may function in a similar way to AtPGB1, as an NO scavenger which inhibits N-degron activity.

The binding site of some ERF-VIIs, referred to as the hypoxia-responsive promoter element (HRPE), is also conserved across species, including tomato (Gasch *et al*., 2016; Reynoso *et al*., 2019). This motif is over-represented in promoters of genes up-regulated by hypoxia or submergence. In Arabidopsis, the HRPE serves primarily as a binding site of the RAP-type ERF-VIIs (Gasch *et al*., 2016), whereas the HRE-type ERF-VII-binding sites are less well studied. AtHRE2 has been shown to bind a tripartite 5′-GCC-3′ element which is distinct from the HRPE but is also enriched in promoters of hypoxia-up-regulated genes (Lee and Bailey-Serres, 2019). This suggests that multiple ERF-VIIs may be regulating expression of hypoxia-regulated genes. Additionally, the HRPE motif itself contains a sequence similar to the 5′-GCC-3′ element (Gasch *et al*., 2016), suggesting that there may be flexibility in which ERF-VII members are able to bind to a particular site. The expression of hypoxia-regulated genes has also been associated with changes in chromatin state, with accessibility of promoters enhanced by hypoxia in both Arabidopsis and tomato (Lee and Bailey-Serres, 2019, 2021).

### Group VII ethylene response factor-independent rapid and systemic low-oxygen stress signals

Besides ERF-VII-mediated transcriptional responses to low-oxygen stress, plants also employ rapid responses that are likely to be independent of the ERF-VII/PCO/N-degron modules. For example, upon waterlogging stress, reactive oxygen species (ROS) change rapidly in plant tissue (Liu *et al*., 2017) and can be transmitted systemically (Peláez-Vico *et al*., 2024). These ROS signals are dependent on RESPIRATORY BURST OXIDASE HOMOLOG D (RBOHD) and can activate redox-specific signaling and biochemical processes (Peláez-Vico *et al*., 2024). In the longer term, low-oxygen stress can also promote ROS production via ERF-VII (RAP2.12) and HYPOXIA RESPONSIVE UNIVERSAL STRESS PROTEIN1 (HRU1) (Gonzali *et al*., 2015). Alternatively, rapid waves in calcium levels facilitated by GLUTAMATE-LIKE RECEPTORS (GLRs) occur during waterlogging stress (Yang *et al*., 2022), allowing systemic root-to-shoot communication, that can be translated to rapid transcriptional changes in leaves (Peláez-Vico *et al*., 2024). These rapid changes (1–10 min) have also been associated with fast hydraulic waves (propagating water pressure) that are linked to fast physiological changes such as stomatal conductance (Peláez-Vico et al., 2024). Similar rapid and systemic turgor pressure changes, calcium waves, and ROS bursts have been linked to other stressors, including wounding and herbivory (Zandalinas *et al*., 2020; Kloth and Dicke, 2022; Grenzi *et al*., 2023). Untangling these rapid systemic responses from the ERFVII-dependent transcriptional responses remains a field of investigation.

## Morphological and anatomical changes in response to flooding

### Aerenchyma formation

**Fig. 2.**
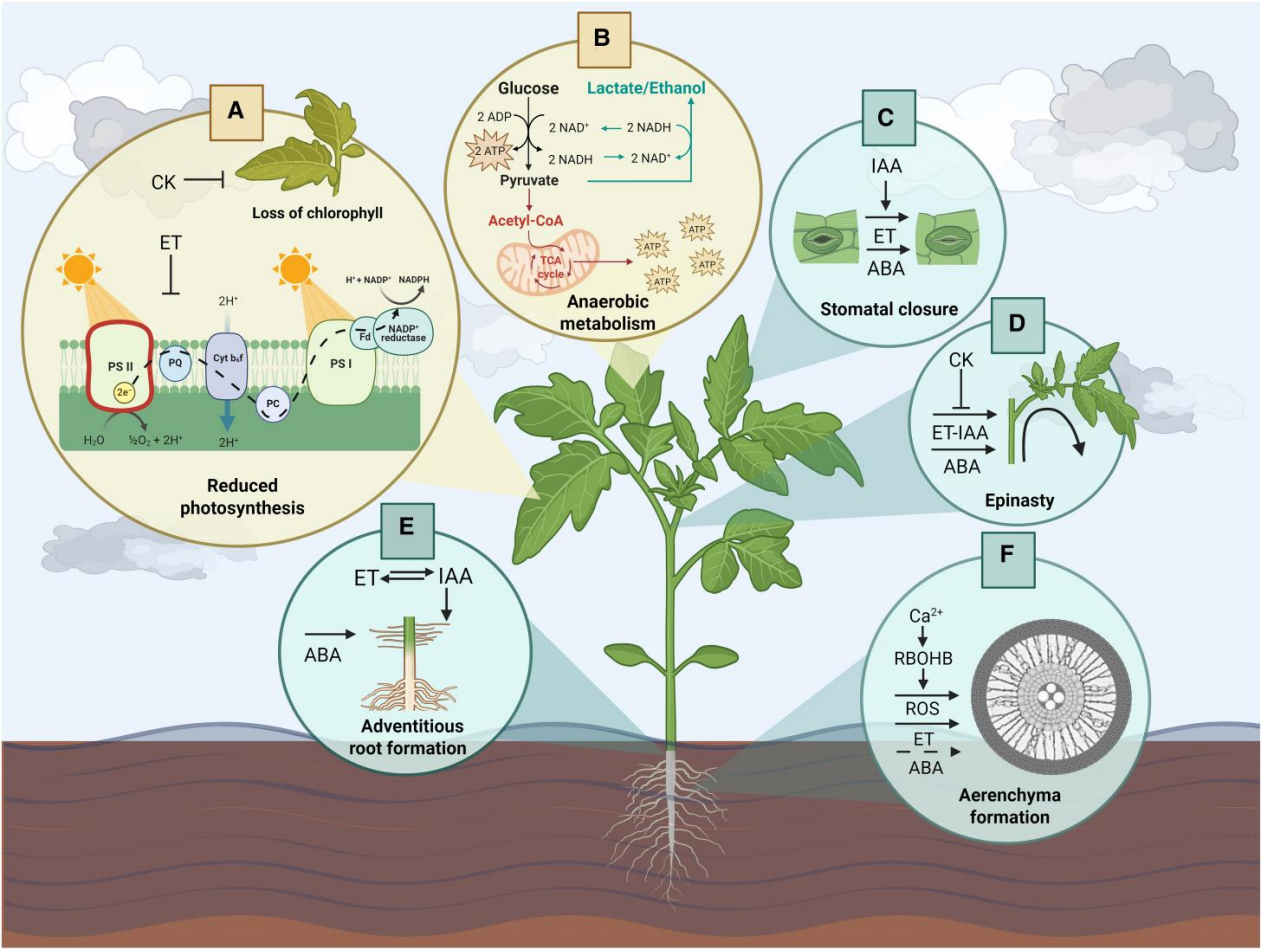
Schematic overview of the morphological and biochemical responses of tomato towards waterlogging stress. (A) Waterlogging impairs photosynthesis by reducing chlorophyll and carotenoid levels. (B) Plants switch to an anaerobic metabolism induced by the hypoxic conditions. (C) Stomatal closure is induced, lowering transpiration rates. (D) Epinasty, or downward leaf bending, is induced to reduce light interception and thus transpiration and photodamage. (E) Emergence of adventitious roots at the stem base to replace damaged roots and aid in recovery. (F) Aerenchyma formation to enhance internal oxygen diffusion. The role of different hormones and signaling molecules is indicated in each process. ET, ethylene; IAA, auxin; ABA, abscisic acid; CK, cytokinins. Created in Biorender. Vanden Broeck, E. (2025) https://BioRender.com/ymc5n71.

Flooding stress markedly reduces gas exchange in submerged organs, making it essential for the plant to develop anatomical changes that facilitate oxygen diffusion through stem and root tissues (Daniel and Hartman, 2024). Several plant species, including tomato, can form aerenchyma (Fig. 2F), which enhances gas movement to and from submerged organs, reduces the number of cells consuming oxygen, and promotes radial oxygen diffusion (Jackson and Armstrong, 1999; Evans, 2004). In tomato, the presence of fragmented cell walls in stem cortical tissue revealed that aerenchyma develops through a lysigenous process (Kawase and Withmoyer, 1980). Cortex porosity becomes evident after 24 h of waterlogging, and large intercellular spaces form within 3 d, with some areas of the epidermis potentially being lost as well (Kawase and Withmoyer, 1980; Mignolli *et al*., 2020). The involvement of ethylene in the formation of lysigenous aerenchyma in stems from flooded tomato plants was confirmed by using AgNO_3_ treatment and the ethylene-insensitive *never ripe* (*nr*) mutant (Mignolli *et al*., 2020).

An RNA-seq analysis conducted by Safavi-Rizi *et al*. (2020) in tomato roots identified genes likely to be involved in aerenchyma formation. One of them is *RBOHB*, which generates superoxide radicals ($O_{2}^{-}$) in the apoplast and is up-regulated in roots during waterlogging. Hypoxia may also facilitate the influx of calcium ions (Ca^2+^) from the apoplast into the cytosol, triggering calcium-dependent kinases that can stimulate RBOHB activity, resulting in increased levels of ROS (Safavi-Rizi *et al*., 2020). Moreover, the expression of genes such as *METALLOTHIONEIN-LIKE 2B* (*MT2B*), encoding ROS scavengers, decreases during waterlogging (Safavi-Rizi *et al*., 2020). This accumulation of ROS probably induces programmed cell death (PCD), facilitating the formation of lysigenous aerenchyma in root and stem cortical cells (Safavi-Rizi *et al*., 2020; Mignolli *et al*., 2020).

The high ethylene levels found in flooded tomato plants are also crucial for the prompt induction of genes encoding cell wall-loosening enzymes in submerged stems. The higher induction of these enzymes causes an increase in cortical cell size, which in turn leads to stem hypertrophy (Mignolli *et al*., 2020). Interestingly, root tissues respond differently to the stress imposed by hypoxia since several genes related to cell wall loosening, including *XYLOGLUCAN ENDOTRANSGLUCOSYLASE/HYDROLASE* and *EXPANSINS*, were down-regulated, particularly at 48 h of treatment (Safavi-Rizi *et al*., 2020).

In tomato, the combined effects of ROS and high ethylene levels lead to PCD and, consequently, to lysigenous aerenchyma formation in submerged tissues. These anatomical modifications enhance oxygen diffusion to submerged organs, promoting metabolic changes such as an increased respiration rate (Mignolli *et al*., 2020). Additionally, some species form a gas barrier (suberization) in the exodermis to limit radial oxygen diffusion (Colmer, 2003; Ejiri *et al*., 2021). In rice and barley, abscisic acid (ABA) has been known to induce a suberized barrier under waterlogging (Shiono *et al*., 2022). In tomato, this suberin deposition has been shown to be controlled by ABA under drought stress (Cantó-Pastor *et al*., 2024). The improved oxygen supply and metabolic activity are essential for supporting the development of adventitious roots, which allow plants to survive even when their original roots perish during waterlogging (Mignolli *et al*., 2020, 2021).

### Adventitious root formation

Adaptation to flooding conditions involves the development of a new root system for most plant species. Indeed, flood-tolerant genotypes of tomato are particularly capable of producing a profuse adventitious root system and increasing hypocotyl porosity (McNamara and Mitchell, 1990) (Fig. 2E). Although periodic waterlogging may limit the growth of adventitious roots (Poysa *et al*., 1987), they can comprise over half of the total root biomass during continuous waterlogging. Furthermore, adventitious roots also play a vital role in helping tomato plants recover from partial submergence. Hilling (piling moist soil at the stem base after draining floodwaters) can promote rapid adventitious root growth, leading to a quicker recovery of root biomass than in non-hilled plants (Mignolli *et al*., 2025). Moreover, hilled plants also restored leaf nitrogen concentration, leaf gas exchange, carbon assimilation, and photochemical efficiency more rapidly than non-hilled plants, pinpointing the important role of adventitious roots during the waterlogging recovery phase (Mignolli *et al*., 2025).

Ethylene is crucial for regulating adventitious root formation in plants, closely interacting with auxins. Ethylene-insensitive plants, such as the natural mutant of ethylene receptor 3 (SlETR3), *nr* (Lanahan *et al*., 1994), exhibit increased below-ground root biomass but reduced adventitious root formation compared with wild-type plants (Clark *et al*., 1999). Conversely, plants with elevated or enhanced ethylene signaling levels show increased adventitious root formation but decreased lateral root development (Clark *et al*., 1999). Indeed, while ethylene generally inhibits lateral root formation and elongation, it promotes adventitious root formation in tomato seedlings (Negi *et al*., 2010). Under flooding conditions, ethylene perception through the NR receptor stimulates auxin transport in roots and auxin accumulation in the stem. This in turn triggers additional ethylene synthesis, creating a positive feedback loop mediated by the *DIAGEOTROPICA* gene, which promotes auxin movement towards the flooded parts of the plant (Vidoz *et al*., 2010). Auxin accumulation at the stem base induces the growth of quiescent adventitious root primordia (Vidoz *et al*., 2010). Inhibiting ethylene biosynthesis or auxin transport reduces adventitious root formation in partially submerged tomato plants, emphasizing the relevance of both hormones in this adaptive response (Vidoz *et al*., 2010).

The *AUREA* (*AU*) gene encodes a phytochromobilin synthase and plays a dual role in chlorophyll production and the formation of adventitious roots, which is mediated by heme homeostasis. A mutation in this gene leads to chlorotic leaves and the spontaneous development of adventitious roots, which help increase submergence tolerance in tomatoes (Wu *et al*., 2020). Grafting experiments suggested that heme may initiate adventitious roots by being transported from leaves to stems. Another mutation affecting adventitious root formation in tomato is observed in the *aerial roots* (*aer*) mutant (Mignolli *et al*., 2017; Kevei *et al*., 2024). This mutant exhibits a profuse formation of adventitious root primordia along the stem, a phenotype linked to altered auxin transport and accumulation. While auxin sensitivity remains unchanged, the *aer* mutant shows an increased response to auxin transport inhibitors and a higher expression of auxin export carrier genes *PIN-FORMED 1* (*PIN1*) and *PIN3* in young tomato seedlings. As *aer* plants mature, there is a significant decrease in auxin efflux and influx gene expression in stems, resulting in slower auxin transport in these tissues and, consequently, in significantly higher levels of free and conjugated indole-3-acetic acid (IAA) at the base of the stems. The altered movement and levels of auxin probably account for the characteristic *aer* phenotype (Mignolli *et al*., 2017). When challenged with waterlogging stress for up to 7 d, the *aer* mutant also displays enhanced tolerance, as it rapidly establishes a new root system that replaces the original one, enabling plants to maintain biomass accumulation comparable with non-flooded conditions (Vidoz *et al*., 2016). The abundance and timing of adventitious root formation and the role of ethylene, auxin, and heme are potential breeding targets for making tomato plants more flood resilient.

### Epinasty

Besides strategies to improve oxygen distribution throughout the plant, tomato has developed another mechanism to reduce damage from waterlogging stress, namely epinasty, characterized by downwards leaf bending (Fig. 2D). Waterlogging-induced epinasty is caused by a higher ethylene production of the shoot (English *et al*., 1995), which activates a hormonal cascade locally triggering differential elongation of the petiole, leading to downward leaf bending or epinasty (Jackson and Campbell, 1976). Epinasty reduces light interception by the plant canopy, which, together with a reduced stomatal conductance, limits the plant transpiration rate (Bradford and Hsiao, 1982; Grichko and Glick, 2001; Else *et al*., 2009). Epinasty also reduces interception of excess light, which limits photodamage, including photoinhibition (Van Geest *et al*., 2012).

Ethylene interacts with downstream pathways, including auxin responses and transport, to induce epinasty (Lee *et al*., 2008; Sandalio *et al*., 2016). While the exact ethylene–auxin crosstalk sequence has long been a matter of debate (Kazemi and Kefford, 1974; Ursin and Bradford, 1989; Keller and Van Volkenburgh, 1997), it was found that ethylene and auxins share transcriptional targets, such as AUXIN/INDOLE-3-ACETIC ACID (AUX/IAA) IAA3, to regulate epinasty (Chaabouni *et al*., 2009). IAA3 facilitates vegetative adaptations throughout development (Chaabouni *et al*., 2009), and is transcriptionally up-regulated during waterlogging (De Ollas *et al*., 2021). In Arabidopsis, expression of *AtIAA3* is partially controlled by TEOSINTE BRANCHED1/CYCLOIDEA/PROLIFERATING transcription factors (TCPs), together with the activation of *miR164* (Koyama *et al*., 2010), an miRNA involved in aging and the ontogenic differentiation of ethylene responses (Kim *et al*., 2009; Li *et al*., 2013). Although the relevance of this IAA3–TCP–*miR164* pathway has not been studied in tomato, ethylene-induced epinasty does depend on leaf age (Edelman and Jones, 2014; Mohorović *et al*., 2024). In addition, the ethylene-mediated initiation of epinasty is ontogenically differentiated through shifts in expression of the *ACC-OXIDASE* (*ACO*) gene family and subsequent ethylene production (De Ollas *et al*., 2021; Geldhof *et al*., 2022).

The developmental and physiological state of a particular leaf is linked to the actual hormone homeostasis, which in turn affects the epinastic response during waterlogging. Young leaves, for example, retain higher levels of cytokinins (CKs) (Geldhof *et al*., 2024) and are able to sustain plasticity in terms of both daily nutations and upward repositioning after reoxygenation (Geldhof *et al*., 2021). An external application of CKs reduces waterlogging-induced leaf epinasty (Jackson and Campbell, 1979; Geldhof *et al*., 2024), yet their mode of action in epinastic bending remains obscure (Eerdekens *et al*., 2024). Besides CKs, other hormones such as ABA have been found to influence the extent and speed of the epinastic response in waterlogged tomato (Geldhof *et al*., 2024) and the hyponastic response in submerged *Rumex palustris* (Cox *et al*., 2004). Furthermore, recent evidence indicates that epinasty is meticulously controlled in time and space by an extensive gene network, giving rise to genotype-specific responses (Geldhof *et al*., 2023).

## Biochemical and physiological responses

### Chlorophyll, photosynthesis, and transpiration effects

Flooding also disrupts plant photosynthesis, being strongly dependent on chlorophyll levels and transpiration rates (Fig. 2A). The photosynthesis pathway requires a functional light-harvesting complex, which contains Chl *a* and Chl *b* pigments and accessory carotenoids (Kühlbrandt *et al*., 1994; Croce *et al*., 1999; Formaggio *et al*., 2001). Flooding stress in tomato results in a reduction in chlorophyll levels (Fig. 2) and, additionally, a decline in carotenoid levels. This decline in pigments will reduce the quantum efficiency of PSII and photochemical quenching, and as such the total quantum yield (Else *et al*., 2009; De Pedro *et al*., 2020; Geldhof *et al*., 2022), which negatively impacts leaf photosynthesis (Fig. 2A) (Else *et al*., 2009; Pan *et al*., 2021; Geldhof *et al*., 2022). The severity of the flooding effect on pigment level seems to be dependent upon a variety of parameters. For example, the duration of the waterlogging event is critical, since pigment levels and chlorophyll fluorescence only change after 24–48 h of stress (De Pedro *et al*., 2020; Safavi-Rizi *et al*., 2020; Geldhof *et al*., 2022). Furthermore, waterlogging seems to have less impact on young tomato leaves compared with older leaves with respect to a decline in chlorophyll content and photosynthesis (Geldhof *et al*., 2022). Cultivar trials showed that waterlogging negatively influences leaf chlorophyll content in many, but not all, genotypes (Kuo and Chen, 1980). The effect of flooding on pigment levels is also impacted by other stressors, such as heat (Lin *et al*., 2016) or repetitive waterlogging stress (Umićević *et al*., 2024). Collectively, these studies show that duration, leaf age, genotype, and environmental conditions render tomato plants more or less susceptible to waterlogging-induced changes in chlorophyll levels and a concomitant drop in photosynthesis.

Besides changes in pigment content, waterlogging stress also induces stomatal closure, leading to a drop in transpiration rate (Pan *et al*., 2021; Yang *et al*., 2023). There seems to be less cultivar dependency with respect to waterlogging-induced reduction in transpiration (Yin *et al*., 2023). It is most likely that ABA is involved in the waterlogging-induced stomatal closure of tomato (Else *et al*., 1996). Repetitive waterlogging stress can prime tomato plants and protect them by adjusting stomatal conductance and photosynthesis (Niu *et al*., 2023; Umićević *et al*., 2024). Overall, waterlogging reduces photosynthesis and transpiration rate, leading to a yield penalty. There is probably an increased sink strength of developing fruit during early phases of waterlogging stress, favoring generative development and fruit ripening (Horchani *et al*., 2009), but long-term waterlogging clearly leads to severe yield losses in tomato (Ide *et al*., 2022; Yin *et al*., 2023).

### Energy metabolism

Besides these physiological effects, flooding also imposes significant metabolic changes. Indeed, among differentially abundant proteins in leaves of waterlogged plants, some were related to energy and sugar metabolism (Ahsan *et al*., 2007; Czernicka *et al*., 2022) (Fig. 2B). Similarly, metabolism- and energy-specific genes were differentially expressed in hypoxic tomato plants (Safavi-Rizi *et al*., 2020; De Ollas *et al*., 2021). The effect of hypoxia on root growth and metabolism varies depending on the developmental stage of the plant, and the balance between source and sink organs and among sink organs themselves. For example, root carbohydrate content decreases significantly when tomato plants undergo gradual root hypoxia, concomitantly with the presence of high sugar-demanding organs such as fruits (Horchani *et al*., 2009). Interestingly, a hypoxic pre-treatment of tomato roots can significantly enhance their tolerance to subsequent anoxia, extending survival from 10 h to >36 h when external sucrose is present (Germain *et al*., 1997). This acclimation process improves the energy metabolism, as evidenced by increased energy charge values in anoxic tissues, and is related to immediate ethanol production under anoxia, with minimal lactic acid accumulation (Germain *et al*., 1997; Czernicka *et al*., 2022). The Davies–Roberts hypothesis proposes that a brief lactate fermentation phase in plant tissues transitioning from normoxic to anoxic conditions triggers ethanol fermentation and inhibits further lactate production through pH changes (Davies *et al*., 1974; Roberts *et al*., 1984). Rivoal and Hanson (1994) overexpressed *LACTATE DEHYDROGENASE* (*LDH*) in tomato roots to test this hypothesis. Despite a 50-fold increase in LDH activity, transgenic roots showed no significant changes in lactate accumulation or fermentative metabolism compared with control plants under normoxic conditions. However, a hypoxic pre-treatment enhanced lactate and ethanol production in both control and transgenic plants, although lactate production notably decreased after the first hour of treatment while ethanol production remained steady. These experiments revealed that LDH has a low level of control of lactate production since both wild-type and transgenic plants behaved similarly. In addition, hypoxia promoted lactate export in transgenic and wild-type roots, which could regulate long-term lactate glycolysis *in vivo*, emphasizing metabolic control in anaerobic conditions (Rivoal and Hanson, 1994). In addition, root hypoxia tolerance relies on effective carbohydrate metabolism, and transgenic tomato plants that overexpress Arabidopsis *HEXOKINASE1* (*AtHXK1*) exhibit an improved energy status (Gharbi *et al*., 2007).

A study on partially submerged tomato plants found that hypocotyls accumulate relatively high amounts of soluble sugars. Surprisingly, while root respiration is suppressed, oxygen consumption increases in hypocotyls (Mignolli *et al*., 2021). This enhanced respiration is likely to be aided by aerenchyma formation in the submerged stem, which helps restore internal oxygen levels. Leaf-derived sucrose accumulates in flooded hypocotyls and is a primary carbon source for respiration. Furthermore, a high sucrose synthase activity in submerged hypocotyls suggests that sucrose is broken down and used for respiration. Notably, inhibiting hypocotyl respiration significantly reduces sugar accumulation, indicating that elevated respiration rates are necessary for sucrose unloading from the phloem. This process creates a positive feedback loop, as increased substrate availability further supports respiration, ensuring continued sugar allocation to flooded hypocotyls (Mignolli *et al*., 2021).

## Hormonal regulation of flooding responses in tomato

It is well established that ethylene plays a pivotal role in various flood-adaptive responses of tomato plants. However, other hormones also contribute, often in crosstalk with each other, to determine local adaptive responses to low-oxygen stress (Geldhof *et al*., 2024). The integration of these hormonal signals is key for activation of flooding responses and, consequently, survival (Fig. 2).

### Ethylene

The flooding-induced restriction of gas exchange influences ethylene diffusion. In the case of submergence, entrapment of ethylene occurs, leading to systemic ethylene responses (Jackson, 2008; Sasidharan and Voesenek, 2015; Leeggangers *et al*., 2023), while waterlogging predominantly enhances accumulation of ethylene in the root zone (Jackson and Campbell, 1976). While root hypoxia impedes the conversion of 1-aminocyclopropane-1-carboxylate (ACC) to ethylene, it also stimulates ACC production (Olson *et al*., 1995; Shiu *et al*., 1998) and its xylem transport to the normoxic shoot (Bradford and Yang, 1980). The increased supply of ACC and its subsequent conversion into either the inactive ACC conjugate malonyl-ACC (MACC) or ethylene will determine the local shoot response (English *et al*., 1995; Geldhof *et al*., 2022). Typically, low oxygen tolerance in many species has been ascribed to ethylene-mediated activation of hypoxia-related gene networks including ETHYLENE RESPONSE FACTORS (ERFs) and, specifically, the group VII ERFs. However, this relationship is not unidirectional in tomato (Hartman *et al*., 2020).

Ethylene acts as a main signal to evoke certain morphological changes to ensure survival, including aerenchyma formation, adventitious root emergence, and epinasty. Ethylene can also inhibit plant photosynthesis (Mohorović *et al*., 2024) and influence sugar and energy metabolism, to activate the quiescence survival strategy during flooding (Hartman *et al*., 2021).

Soluble sugar content decreases in tomato leaves after an ethylene treatment (Mohorović *et al*., 2024). This could in part explain the elevated levels of sugars in the *nr* mutant (Nascimento *et al*., 2021). In addition, the *nr* mutant is able to sustain photosynthetic activity (De Pedro *et al*., 2020) and growth during waterlogging and recovery (De Pedro *et al*., 2020; Geldhof *et al*., 2022).

Crosstalk between ethylene and sugar signaling also provides a possible cue for leaf posture control and epinasty. Impeded ethylene signaling in the *nr* mutant limits epinastic bending and the loss of nutational movements in an ontogenic fashion (Geldhof *et al*., 2022). Similarly, the action of NR is strongly intertwined with downstream pathways such as auxin transport (Negi *et al*., 2010) and signaling (Lin *et al*., 2008). In conclusion, ethylene seems to be a dominant regulator to control leaf epinasty and aerenchyma formation, and modulates other processes such as photosynthesis and sugar metabolism during flooding stress in tomato.

### Auxins

Flooding conditions affect auxin levels and transport, which lead to some plant adaptive responses. In both tomato and sunflower, IAA accumulates more in hypocotyls of flooded plants than in those not flooded (Wample and Reid, 1979; Vidoz *et al*., 2010). This accumulation of auxin triggers the production of ethylene, which further enhances the translocation of auxin toward the submerged parts of the plant, supporting development of adventitious roots (Vidoz *et al*., 2010). In *Rumex palustris*, the endogenous auxin concentration does not significantly change during waterlogging-induced adventitious rooting (Visser *et al*., 1996). However, auxin fluxes from the shoot to the rooting zone should sustain stable IAA concentrations. Although auxin levels remain unchanged during waterlogging, increased ethylene concentrations enhance the sensitivity of root-forming tissues to auxin, promoting the formation of adventitious roots (Visser *et al*., 1996).

Partial submersion enhances the expression of auxin-responsive genes in the adventitious root primordia of *Solanum dulcamara* (Dawood *et al*., 2016). Many of these genes, particularly those encoding LATERAL ORGAN BOUNDARIES DOMAINS (LBD) transcription factors, known to play a role in lateral root development, are up-regulated in adventitious root primordia after 6–24 h of submergence. This indicates that there is an increase in auxin signaling during the activation of these primordia (Dawood *et al*., 2016). However, in the case of complete submersion, disrupting auxin signaling in adventitious root primordia seems critical for impairing the outgrowth of these roots (Yang *et al*., 2018).

Additionally, auxin conjugation plays a role in the escape strategies of deepwater rice cultivars under complete submergence. Two specific auxin conjugates, IAA-Leu and IAA-Asp, decreased in NIL-12 plants, which carry the deepwater response quantitative trait locus (QTL), compared with T65 plants under partial submergence conditions. This reduction in auxin conjugates may be part of the adaptive response to flooding in deepwater rice, indicating that the regulation of IAA-Leu and IAA-Asp is likely to be a crucial element of the metabolic reprogramming that occurs in these varieties as part of their escape strategy during flooding stress (Fukushima *et al*., 2020). The role of auxin conjugation has not yet been studied in waterlogging of tomato.

### Abscisic acid

The involvement of ABA in plant responses to hypoxia stress has been reviewed by González-Guzmán *et al*. (2021) and Zhao *et al*. (2021). It was shown that both hypoxia-induced aerenchyma formation (in soybean, mango, and rice) and stem and petiole elongation (in rice and *Rumex*) are influenced by ABA signaling (Larson *et al*., 1993; Benschop *et al*., 2005; Steffens and Sauter, 2005; Yang and Choi, 2006; Shimamura *et al*., 2014). However, the extent of the role of ABA in *Solanaceae* appears to be rather limited. ABA has been linked to waterlogging-induced stomatal closure in a range of species, including tomato (Neuman and Smit, 1991; Else *et al*., 1996; Gil *et al*., 2009). Upon waterlogging, ABA levels increase in the xylem sap and are mobilized from the root to the shoot (Else *et al*., 1996). Eventually, the concentration of ABA in leaf tissue increases during soil flooding stress, but it remains questionable whether this rise in ABA is causal for stomatal closure, especially because the timing is different (Else *et al*., 1996, 2006). Notably, waterlogging leads to a rapid rise in ABA levels (<12 h) in tomato petioles and young leaves, followed by a gradual decline at later time points in both tissues (Geldhof *et al*., 2024). It is possible that mobile molecules such as nitrate, ROS, and/or NO crosstalk with ABA to close stomata, as they have been implicated in the same process during drought stress in wheat (García-Mata and Lamattina, 2001; Da-Silva and Amarante, 2022), or that ethylene is a secondary messenger that influence the ABA-mediated stomatal closure (Tanaka *et al*., 2008).

Furthermore, the *notabilis* mutant (*not*), having reduced ABA levels, displays a markedly impaired stomatal response to soil flooding, accompanied by an enhancement in transpiration rate (De Ollas *et al*., 2021). Interestingly, ABA levels dropped in root tissue of wild-type tomato plants during waterlogging (De Ollas *et al*., 2021). Similarly, in *S. dulcamara* it was demonstrated that submergence-induced adventitious root formation coincided with a significant decrease in ABA content (Dawood *et al*., 2016). In addition, the application of ABA was shown to inhibit the activation of adventitious root primordia (Dawood *et al*., 2016). Moreover, both the *not* mutant and the ABA transport mutant *ait1.1* have a dampened epinastic bending during waterlogging, suggesting that changes in shoot ABA levels contribute to leaf movements (Geldhof *et al*., 2024). In conclusion, waterlogging reduces root ABA levels, which can stimulate adventitious root development, while ABA levels increase in the shoot, by local synthesis and xylem-mediated transport, where it modulates stomatal closure and epinastic bending.

### Cytokinins

CKs are involved in various physiological processes in plants, including leaf senescence (Glanz-Idan *et al*., 2022). They are also involved in flooding responses, and can modulate stomatal opening, adventitious root formation, leaf senescence, ROS stress, and epinasty (Eerdekens *et al*., 2024). It has been observed that during hypoxia stress, the root-to-shoot transport of CKs is significantly reduced in poplar and common bean (Neuman *et al*., 1990; Smit *et al*., 1990). This was caused by a reduction in the xylem flux and a drop of CK levels in xylem sap, a phenomenon also observed in sunflower (Burrows and Carr, 1969). In tomato, waterlogging causes down-regulation of the *trans*-zeatin-type cytokinin biosynthesis genes *CYP735A1/A2* in roots (Safavi-Rizi *et al*., 2020; De Ollas *et al*., 2021), probably reducing long-distance CK translocation. In fact, waterlogging leads to lower CK levels in petioles and leaves of tomato (Geldhof *et al*., 2024). This waterlogging-induced drop in shoot CK is crucial for establishing the epinastic response in tomato, as the application of a synthetic CK (benzyladenine) inhibits epinastic bending but retains chlorophyll levels and photosynthetic capacity (Railton and Reid, 1973; Jackson and Campbell, 1979; Bradford, 1983). However, the combined application of CKs and gibberellins did not fully restore normal phenotypes during waterlogging, indicative of hormonal crosstalk (Else *et al*., 2009). In conclusion, CK levels are important for normal plant physiology, and a waterlogging-induced drop in CK levels modulates epinastic bending and a concomitant drop in photosynthetic performance.

## Conclusions and future perspectives

As the risk of flooding increases due to climate change, it poses a significant threat to tomato productivity, particularly in uncovered soil-based cultivation systems. While tomato plants exhibit moderate tolerance to flooding events, genotype-specific variations are evident, offering potential for breeding-tolerant varieties. Tomato plants possess the capacity to respond to flooding stress, although the precise mechanisms and regulation of their oxygen signaling pathway remain to be elucidated.

In the first instance, flooded tomato plants will display epinasty and form aerenchyma, two processes predominantly controlled by ethylene, but fine-tuned by auxin, ABA, and CKs. Subsequently, tomato plants alter their sugar and energy metabolism, partly due to oxygen deprivation and reduced photosynthetic performance. The latter coincides with a decrease in stomatal conductance and a loss of chlorophyll pigmentation, processes that can be counteracted by CKs. During extended periods of flooding, tomato plants develop adventitious roots, to facilitate gas exchange and aid in rapid recovery upon reoxygenation. The formation of adventitious roots is primarily regulated by auxins, in crosstalk with ethylene and ABA. This review highlights the diverse and complex regulation of various flooding-adaptive responses in tomato plants, while also providing a scientific foundation for future breeding and biotechnological advancements aimed at enhancing flooding resilience of tomato plants.

Future research endeavors should continue to elucidate the molecular networks governing plant oxygen sensing in tomato. The significance of hormonal crosstalk should be given greater attention in research on flooding stress in tomato and other crops. The advancement of omics approaches and gene-editing technologies holds promise in facilitating the identification and targeted modification of key regulatory genes, thereby enhancing flood tolerance. Furthermore, the integration of physiological and agronomic studies will be essential for the development of climate-resilient tomato cultivars adapted to increasingly variable growing conditions.

## Data Availability

Data will be made available upon a reasonable request.
